# Impact of the Kidney Score Platform on Communication About and Patients’ Engagement With Chronic Kidney Disease Health: Pre–Post Intervention Study

**DOI:** 10.2196/56855

**Published:** 2025-04-29

**Authors:** Delphine Tuot, Susan Crowley, Lois Katz, Joseph Leung, Delly Alcantara-Cadillo, Christopher Ruser, Elizabeth Talbot-Montgomery, Joseph Vassalotti

**Affiliations:** 1 Division of Nephrology, University of California, San Francisco, Zuckerberg San Francisco General Hospital, 1001 Potrero Ave, Bldg 100, Room 342, San Francisco, CA, 94110, United States; 2 Veterans Administration Connecticut Healthcare System, New Haven, CT, United States; 3 Department of Medicine, Yale University, West Haven, CT, United States; 4 Veterans Administration NY Harbor Healthcare System, New York, NY, United States; 5 National Kidney Foundation, New York, NY, United States; 6 Department of Medicine, Icahn School of Medicine, Mt Sinai, New York, NY, United States

**Keywords:** chronic kidney disease, CKD, CKD communication, CKD knowledge, patient activation, kidney, kidney score platform, kidney health, United States, US, adult, aging, clinical practice, awareness campaign, health information, clinician, primary care, longitudinal intervention, web-based, mobile health, mHealth

## Abstract

**Background:**

Chronic kidney disease (CKD) affects 14% of the US adult population, yet patient knowledge about kidney disease and engagement in their kidney health is low despite many CKD education programs, awareness campaigns, and clinical practice guidelines.

**Objective:**

We aimed to examine the impact of the Kidney Score Platform (a patient-facing, risk-based online tool that provides interactive health information tailored to an individual’s CKD risk plus an accompanying clinician-facing Clinical Practice Toolkit) on individual engagement with CKD health and CKD communication between clinicians and patients.

**Methods:**

We conducted a pre-post intervention study in which English-speaking veterans at risk for CKD in two primary care settings interacted with the Kidney Score platform’s educational modules and their primary care clinicians were encouraged to review the Clinical Practice Toolkit. The impact of the Kidney Score on the Patient Activation Measure (the primary outcome), knowledge about CKD, and communication with their clinician about kidney health was determined with paired *t* tests. Multivariable linear and logistic models were used to determine whether changes in outcomes after versus before intervention were influenced by age, race or ethnicity, sex, and diabetes status, accounting for baseline values.

**Results:**

The study population (n=76) had a mean (SD) age of 64.4 (8.2) years, 88% (67/76) was male, and 30.3% (23/76) self-identified as African-American. Approximately 93% (71/76) had hypertension, 36% (27/76) had diabetes, and 9.2% (7/76) had CKD according to the laboratory criteria but without an *ICD-10* (International Classification of Diseases, 10th Edition) diagnosis. Patient interaction with the Kidney Score did not change the mean Patient Activation Measure (preintervention: 40.7%, postintervention: 40.2%, *P*=.23) but increased the mean CKD knowledge score (preintervention: 40.0%, postintervention 51.1%, *P*<.01), and changed the percentage of veterans who discussed CKD with their clinician (preintervention: 12.3%, postintervention: 31.5%, *P*<.01). Changes did not differ by age, sex, race, or diabetes status. Results were limited by the small sample size due to low recruitment and minimal clinician engagement with the Clinical Practice Toolkit during the COVID-19 pandemic.

**Conclusions:**

One-time web-based tailored education for patients can increase CKD knowledge and encourage conversations about kidney health. Increasing patient activation for CKD management may require multilevel, longitudinal interventions that facilitate ongoing conversations about kidney health between patients and clinician teams.

## Introduction

Chronic kidney disease (CKD) affects 37 million Americans [1] and is associated with high risks of emergency department visits, hospitalizations, cardiovascular events, and early mortality [2-5]. Yet, as many as half of all individuals with laboratory manifestations of kidney disease [6] and those at the highest risk of CKD progression to kidney failure [7] are unaware that they have kidney disease. CKD is usually asymptomatic. Individuals cannot readily know their disease status or risk for disease without risk recognition, testing, detection, and communication by clinicians [8]. A clinician diagnosis of CKD has been associated with increased delivery of evidence-based care, as well as increased patient awareness of their kidney disease [9-11]. However, clinician detection of CKD and communication about kidney disease in the United States are suboptimal [12,13].

Interventions to enhance patient CKD knowledge, increase patient engagement in kidney health, and promote effective communication about CKD between patients and clinicians are critical to facilitate the identification of high-risk populations that would benefit the most from aggressive management of CKD and nephrology referral. They are also important for individuals at a lower risk of CKD progression, to ensure accurate drug dosing, avoid nephrotoxic medications, and encourage the optimal management of diabetes and hypertension, the two most common etiologies of kidney failure in the United States [14].

Existing education programs, awareness campaigns, and clinical practice guidelines have minimally improved CKD awareness in the US population [15,16]. One reason may be because interventions have targeted either the patient or the provider, but rarely both at the same time. To bridge this gap, the National Kidney Foundation developed the Kidney Score Platform, which includes a patient-facing, risk-based online tool that provides interactive health information tailored to an individual’s CKD risk as well as an accompanying clinician-facing Clinical Practice Toolkit [17]. The patient-facing elements aim to increase individual awareness of CKD and encourage patients with and at risk for CKD to initiate discussions about kidney disease with their clinicians. The Clinical Practice toolkit assists clinicians in discussing CKD with individuals with and at risk for kidney disease. In this manuscript, we describe the impact of the Kidney Score Platform on individual engagement with their CKD health and CKD communication between clinicians and patients.

## Methods

### Study Design, Population, and Settings

This was a pre-post–intervention study that examined the impact of the Kidney Score on CKD knowledge and participation in CKD self-management. The Kidney Score is a web-based educational platform designed to improve awareness and understanding of kidney disease among individuals at risk and living with CKD. The study took place in the primary care settings of the Veterans Affairs Medical Centers (VA New York Harbor Healthcare System and VA Connecticut Healthcare System) between September 2022 and March 2023. Eligible participants included English-speaking veterans between the ages of 18 and 75 years with diabetes or hypertension defined in the electronic health record (EHR). The exclusion criteria included an estimated glomerular filtration rate (eGFR) <15 ml/min/1.73 m^2^ or individuals with end-stage kidney disease or kidney transplant recipients, as the Kidney Score is not geared towards individuals with severe CKD. Veterans enrolled in hospice services, those with vision impairment, and those with severe dementia identified in the EHR or during the consent process were also excluded from the study, as CKD awareness is much less important for this population’s overall health.

### Ethical Considerations

Eligible veterans with an upcoming primary care visit at one of two participating Veterans Affairs Medical Centers (VA New York Harbor Healthcare System and VA Connecticut Healthcare System) were identified by the study team, leveraging the EHR. These veterans were invited to participate in the study by mail. Individuals who did not opt out were contacted by phone; those that provided telephone assent were mailed/emailed an information sheet describing the program. During a subsequent phone call, verbal informed consent was obtained and participants were asked to complete an online preintervention survey administered by the study team. Study participants were given a unique study ID; all collected data were deidentified. Participants were given a US $30 gift card for participating. The study was approved by the Institutional Review Boards of the VA Connecticut Healthcare System (#02290) and VA NY Harbor Healthcare System (#01705).

### Study Processes and Intervention

The Kidney Score is a free, online website that uses a rule engine and risk predictive analytics to provide interactive health information tailored to an individual’s CKD risk and health status. In an anonymous fashion, individuals enter risk factor information as well as laboratory findings in the Kidney Score Platform’s online interface and receive educational programming tailored to their clinical status and risk for CKD development or progression. Development of the Kidney Score has been previously described in detail and leveraged the Behavioral Change Wheel framework [18]. It was subsequently found to be acceptable to patients with and at risk for kidney disease [17].

After providing informed consent and baseline data, study participants were invited by the study team to engage with the Kidney Score Platform, which included educational modules tailored to their CKD risk as determined by the Kidney Score’s rule engine. Veterans were given the option to print out questions to ask their providers about their kidney health and risk for kidney disease. Participants could access the tool in any location of their preference (ie, home, waiting room, or community center). Within a week after completing their subsequent primary care appointment, participants were asked to complete a postintervention closed survey electronically to assess the impact of the Kidney Score on their understanding of CKD and the quality of their conversation with their clinician about kidney health.

The pre- and postintervention surveys consisted of 4 instruments and 1 additional question. The question order was fixed for all the study participants; the surveys spanned 5 screens and did not include any adaptive components. Survey responses were all voluntary; there was no “completeness check” and participants were allowed to “go back” to change survey responses prior to submitting the final data.

### Outcomes

The primary outcome was the change in self-efficacy for CKD management, ascertained by the Patient Activation Measure [19] after the participant engaged with the Kidney Score and completed a visit with their primary care clinician compared to preintervention. Secondary outcomes included self-reported changes in CKD knowledge [20], perceived risk of kidney disease [21], and communication quality with providers [22], all ascertained by validated instruments. The change in the percentage of individuals who discussed kidney disease with their provider was also assessed using the question “At your last visit with your primary care clinician, did you discuss your risk for chronic kidney disease or testing for kidney disease?”

### Measurements

Patient demographic data (ie, age, sex, and race or ethnicity) were self-reported by participants. Prevalent comorbid conditions (diabetes, hypertension, and CKD) and the baseline eGFR were ascertained from the EHR.

### Statistical Analysis

Power calculations performed before study activities suggested that 103 individuals would need to participate in the study to detect a clinically meaningful change in the Patient Activation Measure (the primary outcome). Due to challenges with study operations with the transition towards virtual care delivery during the COVID-19 pandemic, the study was stopped after the full participation of 76 individuals. We examined the characteristics of these study participants using descriptive statistics. Changes in the Patient Activation Measure, communication quality with providers, and kidney knowledge were determined with paired *t* tests. The McNemar test for binary paired data was used to ascertain changes in the perceived risk of kidney disease and the concern for developing kidney disease. Multivariable linear models (for continuous outcomes) and logistic models (for dichotomous outcomes) were used to determine whether changes in outcomes post versus preintervention were influenced by age, race or ethnicity, sex, and diabetes status, after accounting for baseline values. Statistical analyses were performed using Stata/SE, version 14.0 (StataCorp).

## Results

**Figure 1.**
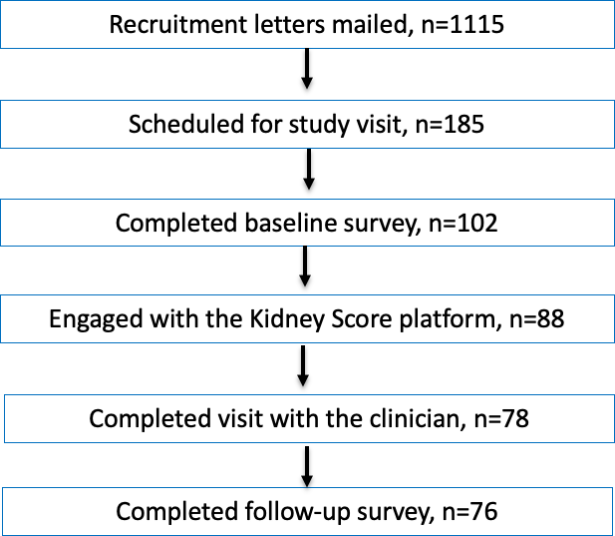
CONSORT (Consolidated Standards of Reporting Trials) diagram for this implementation study assessing the impact of the kidney score on chronic kidney disease knowledge and participation in self-management.

Recruitment letters were sent to 1115 eligible veterans, of whom 87 (7.8%) opted out of receiving further communication. The study team was able to contact 206 veterans by phone, of whom 185 agreed to participate (Figure 1). Reasons for declining participation included being uncomfortable with online technology, mistrust of the study team, mistrust in the Veterans Affairs treatment center, not being interested in kidney disease, and not having the time to participate in a study. Approximately 41% (76/185) of the recruited veterans completed all aspects of the study, including completing the previsit survey, reviewing the web-based Kidney Score Platform, attending a scheduled visit with their primary care clinician, and completing the postvisit survey. The study population had a mean (SD) age of 64.4 (8.2) years and 88% (67/76) were male. Nearly one-third (23/76) of the study participants self-identified as African-American and 5% (4/76) self-identified as Native Hawaiian, Pacific Islander, or Asian. Approximately 93% (71/76) had hypertension and 36% (27/76) had diabetes; 9.2% (7/76) of the population had CKD defined by an eGFR 15‐60 ml/min/1.73 m^2^ but none had a diagnosis of CKD in their clinical record (Table 1). Characteristics of the study population were qualitatively similar to those describing the overall eligible primary care population in the participating clinics (Multimedia Appendix 1) .

**Table 1.** Characteristics of veterans with and at risk for chronic kidney disease (CKD) who participated in this study that examined the impact of the kidney score on CKD knowledge and participation in CKD self-management.

Characteristics	N=76
Age, years, mean (SD)	64.4 (8.2)
Male sex, n (%)	67 (88.2)
Race, n (%)	
American Indian or Alaskan Native	1 (1.3)
Asian	2 (2.6)
Black	23 (30.3)
Native Hawaiian or Pacific Islander	2 (2.63)
Declined to answer	5 (6.6)
White	43 (56.6)
Ethnicity, n (%)	
Hispanic or Latino	4 (5.3)
Not Hispanic or Latino	71 (93.4)
Declined to answer	1 (1.3)
Hypertension,^a^ n (%)	71 (93.4)
Diabetes,^a^ n (%)	27 (35.5)
Chronic kidney disease^b^	7 (9.2)

a Hypertension and diabetes are defined by the *ICD-10* (International Classification of Diseases, 10th Edition) code.

b Chronic kidney disease is defined by an estimated glomerular filtration rate of 15‐60 ml/min/1.73 m2.

Among veterans with complete data, the mean (SD) Patient Activation Measure score at baseline was 40.7% (5.32%). After interacting with the Kidney Score Platform and attending a visit with their provider, the mean Patient Activation Measure score was 40.2% (5.48%), which was largely unchanged (*P*=.23). Similarly, the self-reported communication quality with providers did not change after the interaction with the Kidney Score Platform, from a mean (SD) baseline score of 2.77 (1.06) out of 3.00 to a mean score of 2.84 (0.94; *P*=.51). On multivariable regression models, age, sex, race or ethnicity, and diabetes status were not associated with changes in the Patient Activation Measure scores nor the communication quality.

**Figure 2.**
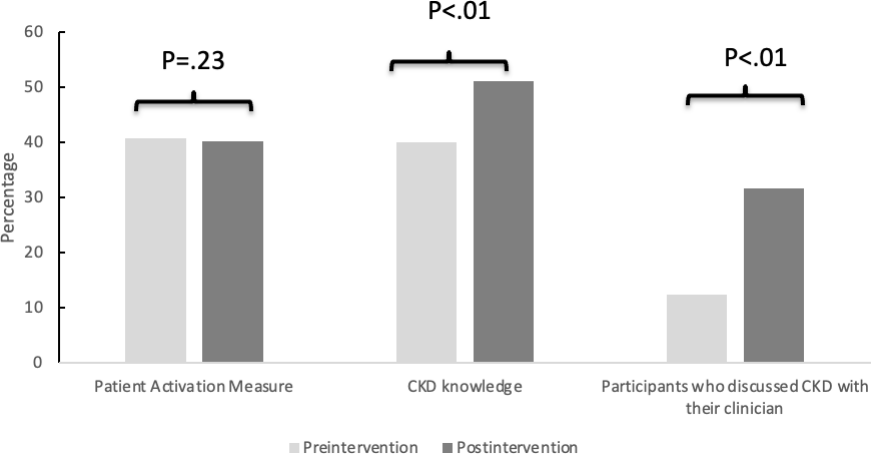
Change in individual behaviors potentially associated with self-efficacy for chronic kidney disease (CKD) management after engagement with the kidney scope online platform.

The mean (SD) CKD knowledge score increased after the intervention from a baseline of 40% (25) to 51.1% (26.5), representing a statistically significant difference (*P*<.01). Similarly, the percentage of veterans who discussed CKD with their clinician in their prior visit increased from 12.3% at baseline to 31.5% after interacting with the Kidney Score Platform (*P*<.01). In multivariable regression models, change in CKD awareness and communication about CKD did not differ by age, sex, race, or diabetes status (Figure 2).

Consistent with the increased communication about CKD in a low-risk primary care population, the proportion of individuals with substantial concern for developing kidney problems or kidney disease in the next 10 years did not change significantly after participating in the study. At baseline, an estimated 52.1% of individuals reported feeling moderately or very concerned for developing CKD; after the study, this decreased to an estimated 42.3% (*P*=.06). Similarly, the estimated percentage of individuals who reported a moderate or high likelihood of developing kidney disease in the next 10 years did not change substantially before versus after the intervention (29.6% vs 31.0%; *P*=.7). Age, sex, race, and diabetes were not associated with greater concern or perceived risk of developing kidney disease.

## Discussion

### Principal Findings and Comparison With Previous Work

The Kidney Score Platform is one of the first interventions that has successfully increased patient knowledge about CKD and communication about kidney health between primary care clinicians and patients. This success can be attributed to its development based on the Behavioral Change Wheel [23], a well-recognized individual behavioral change theory. Theory-informed interventions are more likely to be effective and sustainable within the context of health care delivery than those that are not. The development of the Kidney Score Platform was based on extensive formative work, including qualitative interviews with clinicians and patients [18], as well as proto-typing, beta-testing, and usability testing with various stakeholders, culminating in a refined educational patient experience [17]. This process identified the importance of a tool with which both patients and clinicians could interact and strengthen their communication skills around kidney disease and kidney health.

Despite an increase in CKD conversations and CKD knowledge, the Kidney Score Platform did not increase patient activation for CKD management, which is a hallmark of self-efficacy. The baseline mean (SD) Patient Activation Measure score was 40.1% (5.3%), representing level 1 activation, characterized by individuals who may not believe that the patient’s role is important in chronic disease management. This reflects a large opportunity for improvement, rendering this study’s null primary outcome results disappointing. We speculate that the lack of change in the Patient Activation Measure score can be attributed in part to the suboptimal sample size, as well as the characteristics of the study population and the intervention itself. Veterans who participated in this study were primarily at risk for CKD (69/76) with only 9.2% (7/76) having laboratory evidence of CKD considering the eGFR. Engaging with the Kidney Score may be more motivating among those with CKD versus those at risk for CKD. Similarly, clinician discussions about CKD and how individuals can participate in self-management are likely more impactful for individuals with laboratory evidence of kidney disease compared to those at risk. The absence of a change in the perceived risk of kidney disease among study participants supports this lack of activation among those at risk for CKD. While we did not have the statistical power to assess changes in patient activation by CKD status, we suspect that a study population enriched with veterans with CKD (as opposed to those at risk for CKD) would have led to a greater change in patient activation.

The nature of the intervention may have also contributed to a lack of change in patient activation. While a handful of patient interactions with the Kidney Score Platform and one patient-initiated discussion with a clinician during a routine primary care visit may be sufficient to increase the broad understanding of kidney disease among individuals with or at risk for CKD, it is likely insufficient to motivate the need for behavior change to optimize kidney health. More than one conversation about kidney disease is likely needed to enhance participation in healthy behaviors and promote self-confidence in other aspects of CKD self-management. With that in mind, it is possible that patient activation was remeasured too early after the time-limited intervention. While speculative, it is plausible that an initial interaction with the Kidney Score Platform may have prompted future discussions about kidney disease and thus future changes in patient activation. Future interventions leveraging the Kidney Score Platform or other patient-facing educational modules will need to include multiple opportunities for discussions about kidney health over time, while also being mindful to not exacerbate potential health anxiety associated with greater knowledge about kidney disease. This is consistent with Kolb’s theory of experiential learning, which posits that most adult learning is gained through experience and engagement over time [24].

The application of Kolb’s theory has been demonstrated for the CKD population with moderate to severe CKD depicted by an eGFR 15‐60 ml/min/1.73 m^2^. For example, Medicare’s Kidney Disease Education program is designed to be delivered in 6 sessions for those with an eGFR 15‐30 ml/min/1.73 m^2^ and includes modules about knowledge about risk factor control, how the kidneys function, medication management, nutrition, and treatments for kidney failure [25]. Two recent studies demonstrated improvements in access to home dialysis and improved preparation for hemodialysis for the population that received kidney disease education versus eligible controls, although the education delivery was low, among approximately 1% (3469/369,968) of eligible patients [26,27]. A systematic review of educational interventions for patients with CKD with an eGFR range similar to our study participants found that more frequent interventions, (ie, weekly or monthly) were more effective [28]. The elements of the Kidney Score such as the website interaction could easily be scaled for more frequent interventions in future educational investigations.

Additionally, while this study was originally designed as a multilevel intervention directed at patients and clinicians, challenges during the COVID-19 pandemic mitigated the clinician focus of this study. Clinician input refined the Kidney Score Platform, but engagement with the Clinical Practice Toolkit was minimal due to clinician burn out and an emphasis on COVID-19 during the study period. The intervention was ultimately patient-focused with little emphasis on changing clinician behaviors with respect to discussing CKD with individuals with and at risk for kidney disease. Given the strong consensus that multilevel interventions are needed to address health communication [29], future research is needed on what types of tools can be integrated into clinician workflows without exacerbating burn out.

### Strengths and Limitations

The strengths of this investigation include theory-informed patient-focused education to improve multiple domains of patient knowledge and engagement assessed using validated instruments. The limitations include low recruitment that may have biased results to the null, necessary study design refinement during the COVID-19 pandemic, absence of a quantitative measure identifying the number of times a participant engaged with the Kidney Score Platform, and limited generalizability to nonveterans considering the administration of the health care delivery systems.

### Conclusions

Educating, engaging, and empowering individuals at risk for and living with CKD are critical. The findings of this study suggest that web-based tailored education for patients can increase CKD knowledge and encourage conversations about kidney health. Increasing patient activation and self-efficacy for CKD management will require multilevel, longitudinal interventions that facilitate ongoing conversations about kidney health between patients and clinician teams.

## Supplementary material

10.2196/56855 Multimedia Appendix 1 Characteristics of the primary care population potentially eligible for the study, March 1, 2020 to February 28, 2022.

## Abbreviations

CKD: chronic kidney disease
eGFR: estimated glomerular filtration rate
EHR: electronic health record
