# Supplementary material for: Impact of the Kidney Score Platform on Communication About and Patients’ Engagement With Chronic Kidney Disease Health: Pre–Post Intervention Study
Source: JMIR Form Res. 2025 Apr 29;9:e56855. doi: 10.2196/56855 (PMC12054969; doi:10.2196/56855)
Supplement: Multimedia Appendix 1 [file formative-v9-e56855-s001.docx]

**Supplemental Material**

| **Supplemental Table: Characteristics of the primary care population potentially eligible for the study, March 1 2020 - February 28 2022.** | | |
| --- | --- | --- |
|  | **VA Connecticut Healthcare System** | **VA New York Harbor Healthcare System** |
| N | 17458 | 12556 |
| Age, mean (SD) | 62.9 ± 11.9 | 61.1 ± 12.3 |
| Male gender, n (%) | 16204 (93%) | 11163 (89%) |
| Race, n (%) |  |  |
| American Indian or Alaska Native | 73 (0%) | 45 (0%) |
| Asian | 82 (0%) | 305 (2%) |
| Black | 2853 (16%) | 6289 (50%) |
| Native Hawaiian or Pacific Islander | 97 (1%) | 140 (1%) |
| Unknown | 655 (4%) | 833 (7%) |
| White | 13698 (78%) | 4944 (39%) |
| Ethnicity, n (%) |  |  |
| Hispanic or Latino | 1067 (6%) | 2357 (19%) |
| Not Hispanic or Latino | 14530 (83%) | 9985 (80%) |
| Unknown | 1861 (11%) | 214 (2%) |
| Hypertension, n (%) | 16773 (96%) | 12023 (96%) |
| Systolic blood pressure, mean (SD) mm Hg | 137.3 ± 18.0 | 133.0 ± 17.1 |
| Diastolic blood pressure, mean (SD) mm Hg | 75.9 ± 10.8 | 80.3 ± 9.8 |
| Diabetes, n (%) | 5557 (32%) | 4315 (34%) |
| Glycosylated hemoglobin, mean (SD) | 6.1 ± 1.2 | 6.4 ± 1.4 |
| Atherosclerotic cardiovascular disease, n (%) | 4241 (24%) | 2703 (22%) |
| Heart Failure, n (%) | 973 (6%) | 908 (7%) |
| Serum creatinine, mean (SD) mg/dL | 1.1 ± 0.3 | 1.1 ± 0.3 |
| eGFR, mean (SD) ml/min/1.73m^2^ | 80.1 ± 19.0 | 81.7 ± 19.9 |
| uACR, median (IQR) mg/g | 16.0 (7.0-51.0) | 15.3 (5.3-64.1) |
| Nephrology visit, n (%) | 793 (5%) | 656 (5%) |
| Due to missing data, N for systolic and diastolic blood pressure = 16207 and 11735; N for glycosylated hemoglobin = 15099 and 10521; N for serum creatinine and eGFR = 16594 and 11706 | | |
| Abbreviations: SD = standard deviation; eGFR = estimated glomerular filtration rate; uACR = urine albumin-to-creatinine ratio | | |
